# Supplementary material for: A Retrospective Chart Review of Factors Impacting Psychotropic Prescribing Patterns and Polypharmacy Rates in Youth with Autism Spectrum Disorder during the COVID-19 Pandemic
Source: J Clin Med. 2022 Aug 18;11(16):4855. doi: 10.3390/jcm11164855 (PMC9410032; doi:10.3390/jcm11164855)
Supplement: Supplementary file 1 [file jcm-11-04855-s001.zip › jcm-1808797-supplementary.pdf]

Supplementary Materials

Table S1. Indication Based Medication Classes and Examples.

Indication Based Medication Classes	Examples of Medications Utilized in Study
ADHD Medications	amphetamine-dextroamphetamine salt preparations, methylphenidate preparations, clonidine, guanfacine, atomoxetine
Antidepressants	sertraline, escitalopram, fluoxetine, fluvoxamine, duloxetine, venlafaxine, clomipramine, bupropion, mirtazapine, and vortioxetine
Mood Stabilizers	lithium, lamotrigine, carbamazepine, oxcarbazepine, and valproic acid
Anxiolytics	alprazolam, clonazepam, diazepam, lorazepam, buspirone, propranolol, and hydroxyzine
Hypnotics	zolpidem, triazolam, doxepin, trazodone, and prazosin
Antipsychotics	haloperidol, chlorpromazine, risperidone, quetiapine, olanzapine, lurasidone, aripiprazole
Excluded medications	melatonin, diphenhydramine, N-acetylcysteine, benztropine, trihexyphenidyl, cyproheptadine, or metformin

Table S2. Number of Visits, Unique Med, and Med Class per year of the total clinic cohorts.

		Number of Visit	Number of Unique Meds	Number of Classes
2019 (N = 851)	Mean (SD)	2.99 (1.89)	2.58 (1.63)	2.00(1.13)
Frequency	0	NA	5.2 %	5.2 %
	1	25.7 %	23.3 %	32.9 %
	2	21.3 %	26.2 %	32.0 %
	3	20.9 %	19.2 %	19.3 %
	4 or more	32.1 %	26.2 %	10.7 %
2020 (N = 525)	Mean (SD)	3.13 (2.04)	2.62 (1.40)	2.10 (1.02)
Frequency	0	NA	0 %	0.2 %
	1	21.7 %	24.4 %	32.0 %
	2	23.2 %	28.6 %	37.3 %
	3	23.0 %	20.8 %	20.8 %
	4 or more	32.0 %	26.3 %	9.7 %

NA: Not applicable.

Table S3. Number of Visits, Unique Med, and Med Class between not-paired vs paired sample in 2019.

		Number of Visit	Number of Unique Meds	Number of Classes
Not paired (N = 378)	Mean (SD)	2.35 (1.66)	2.16 (1.54)	1.74 (1.15)
Frequency	0	NA	10.6 %	10.6 %
	1	39.9 %	30.4 %	38.9 %
	2	24.1 %	21.7 %	26.5 %
	3	18.0 %	18.5 %	15.1 %
	4 or more	18.0 %	18.8 %	9.0 %
Paired (N = 473)	Mean (SD)	3.50 (1.91)	2.92 (1.61)	2.20 (1.07)
Frequency	0	NA	0.8 %	0.8 %
	1	14.4 %	17.5 %	28.1 %
	2	19.0 %	29.8 %	36.4 %

	3	23.3 %	19.7 %	22.6 %
	4 or more	43.3 %	32.1 %	12.1 %

NA: Not applicable.

Table S4. Number and percentage of patients receiving psychotropic polypharmacy (PP) organized by demographic factors.

Year	Ages	No of Patients	% Patients on PP	*p* Value
2019	3 to 5 years	7	38.8	0.002
	6 to 12 years	255	68.7	
	13 to 17 years	244	73.4	
	18 to 21 years	123	78.3	
2020	3 to 5 years	2	33.3	0.067
	6 to 12 years	153	78.5	
	13 to 17 years	162	78.6	
	18 to 21 years	80	73.4	
Year	Sex	No of patients	% patients on PP	*p* value
2019	Female	124	73.8	0.471
	Male	485	71	
2020	Female	91	79.1	0.321
	Male	306	74.6	
Year	Ethnicity	No of patients	% patients on PP	*p* value
2019	Non-Hispanic	506	72.5	0.338
	Hispanic	62	65.2	
	Unknown	41	70.7	
2020	Non-Hispanic	341	76.3	0.466
	Hispanic	35	68.6	
	Unknown	21	77.8	
Year	Race	No of patients	% patients on PP	*p* value
2019	White	395	73.6	0.102
	Other	90	64.2	
	Asian	46	66.6	
	2 or more	44	68.8	
	Black	34	80.9	
2020	White	257	75.5	0.638
	Other	53	74.6	
	Asian	34	69.4	
	2 or more	31	83.8	
	Black	22	78.6	
Year	Insurance	No of patients	% patients on PP	*p* value
2019	Commercial	404	74.1	0.083

	Medicaid	187	66.8	
	Other	18	69.2	
2020	Commercial	255	75.2	0.954
	Medicaid	133	76.4	
	Other	9	75	

Percentage of patients calculated based on the the number of patients who exhibited PP within each demographic subclassification. For example, in 2019 74.1% of commercially insured patients and 80.9% of patients who identify as Black exhibited PP.

Table S5. Number and percentage of patients receiving multiclass psychotropic polypharmacy (MPP) organized by demographic factors.

**Year**	**Ages**	**No of Patients**	**% Patients on MPP**	***p* Value**
2019	3 to 5 years	5	27.7	<0.001
	6 to 12 years	205	55.2	
	13 to 17 years	200	65.6	
	18 to 21 years	117	74.5	
2020	3 to 5 years	2	33.3	0.046
	6 to 12 years	128	62.7	
	13 to 17 years	149	72.3	
	18 to 21 years	77	70.6	
Year	Sex	No of patients	% patients on MPP	*p* value
2019	Female	112	66.6	0.158
	Male	415	60.7	
2020	Female	81	70.4	0.495
	Male	275	67.1	
	Ethnicity	No of patients	% patients on MPP	*p* value
2019	Non-Hispanic	439	62.9	0.395
	Hispanic	53	55.8	
	Unknown	35	60.3	
2020	Non-Hispanic	304	68.1	0.579
	Hispanic	32	62.7	
	Unknown	20	74.1	
Year	Race	No of patients	% patients on MPP	*p* value
2019	White	344	64.1	0.298
	Other	79	56.4	
	Asian	38	55	
	2 or more	38	59.4	
	Black	28	66.7	
2020	White	230	67.6	0.801
	Other	49	69	
	Asian	30	61.2	

	2 or more	27	72.9	
	Black	20	71.4	
Year	Insurance	No of patients	% patients on MPP	*p* value
2019	Commercial	351	64.4	0.128
	Medicaid	162	57.9	
	Other	14	53.8	
2020	Commercial	232	68.4	0.406
	Medicaid	118	67.8	
	Other	6	50	

Percentage of patients calculated based on the the number of patients who exhibited MPP within each demographic subclassification. For example, in 2019 64.4% of commercially insured patients and 66.7% of patients who identify as Black exhibited MPP. .

Table S6. Medication classes prescribed in 2019 and 2020.

	**2019**	**2020**
	N [1] (%)	N (%)
ADHD Medication	569 (33.5)	370 (33.5)
Antidepressants	484 (28.5)	330 (29.9)
Antipsychotics	273 (16.1)	168 (15.2)
Anxiolytics	184 (10.8)	117 (10.6)
Mood stabilizers	124 (7.3)	78 (7.1)
Hypnotics	65 (3.8)	40 (3.6)
Total [2]	1699 (100)	1103 (100)

[1] indicates the number of subjects who received at least one prescription classified into one of the 6 classes; [2] indicates total number of patients who received at least one prescription to each medication classes.

Table S7. Most prescribed unique medications in 2019 and 2020.

	**2019**	**2020**
	N [1] (%)	N (%)
methylphenidate	235 (10.7)	140 (10.2)
guanfacine	230 (10.5)	157 (11.4)
sertraline	205 (9.3)	125 (9.1)
clonidine	182 (8.3)	125 (9.1)
fluoxetine	144 (6.6)	101 (7.3)
risperidone	139 (6.3)	80 (5.8)
aripiprazole	96 (4.4)	55 (4.0)
hydroxyzine	95 (4.3)	69 (5.0)
Total [2]	2198	1378

[1] indicates the number of subjects who received at least one psychotropic prescription; [2] indicates total number of patients who received at least one prescription for psychiatric medication.
